# Characterization of the diversity, genomic features, host bacteria, and distribution of crAss-like phages in the pig gut microbiome

**DOI:** 10.3389/fvets.2025.1582122

**Published:** 2025-04-22

**Authors:** Yaxiang Wang, Chao Wei, Zhe Chen, Mengqing Zhou, Lusheng Huang, Congying Chen

**Affiliations:** National Key Laboratory of Pig Genetic Improvement and Germplasm Innovation, Jiangxi Agricultural University, Nanchang, China

**Keywords:** crAss-like phage, pig, gut, genomic features, host bacteria, *Prevotella copri*

## Abstract

Phages play an important role in shaping the gut microbiome. CrAss-like phages, which are key members of the gut virome, show high abundance in the human gut and have attracted increasing interest. However, few studies have been found in pigs, and the distribution of crAss-like phages across broader pig populations remains unknown. Here, we obtained 1,251 pig crAss-like phage genomes from 403 metagenomes publicly available and a pig gut virome dataset constructed by ourselves. These crAss-like phage genomes were further clustered into 533 virus operational taxonomic units (vOTUs). Phylogenetic analysis revealed that crAss-like phages in pig guts were distributed across four well-known family-level clusters (Alpha, Beta, Zeta, and Delta) but were absent in the Gamma and Epsilon clusters. Genomic structure analysis identified 149 pig crAss-like phage vOTUs that utilize alternative genetic codes. Gene blocks encoding replication and assembly proteins varied across crAss-like phage clusters. Approximately 64.73% of crAss-like phage genes lacked functional annotations, highlighting a gap in understanding their functional potential. Numerous anti-CRISPR protein genes were identified in crAss-like phage genomes, and CAZymes encoded by these phages were primarily lysozymes. Host prediction indicated that bacterial hosts of pig crAss-like phages primarily belonged to *Prevotella*, *Parabacteroides*, and *UBA4372*. We observed that interactions between crAss-like phages and *Prevotella copri* might have a possible effect on fat deposition in pigs. Finally, all detected vOTUs exhibited low prevalence across pig populations, suggesting heterogeneity in crAss-like phage compositions. This study provides key resources and novel insights for investigating crAss-like phage-bacteria interactions and benefits research on the effects of crAss-like phages on pig health and production traits.

## 1 Introduction

The virome has been considered as “dark matter” in the microbiome due to the lack of comprehensive study. Recent years, with the development of metagenomic sequencing technologies and bioinformatic tools, it has become one of the hot research topics in the microecosystem area (1–3). Various evidences have indicated that phages can shape the gut microbiome through a variety of ways, such as lysing host bacteria, integrating into host bacteria as proviruses, and metabolic reprogramming *via* auxiliary metabolic genes (AMGs) (4, 5). Among the growing datasets of viruses, crAss-like phages are one of the most notable new viral members that are extremely widespread with high abundance in the human gut (6).

Dutilh et al. (7) assembled the first crAss-like phage genome in human fecal metagenomes with a circular genome of ~97 kb in length. Since then, a growing number of crAss-like phages have been identified through different studies (8–10). Guerin et al. (10) classified 249 crAssphage genomes into four clusters of Alphacrassvirinae, Betacrassvirinae, Gammacrassvirinae, and Deltacrassvirinae according to the percentage of shared homologous proteins. Subsequently, the International Committee on Taxonomy of Viruses (ICTV) officially defined the order Crassvirales which include four families and ten subfamilies based on the phylogenetic tree of conserved viral genes (11). Yutin et al. (9) discovered two additional potential subclusters of crAss-like phages at the family level, which were defined as Zeta and Epsilon groups. These crAss-like phages have been detected not only in the human guts, but also in the guts of many other animals. For instance, Edwards et al. (8) found a series of nearly complete genomes of distant relatives of crAss-like phages in non-human primates. Additionally, Shkoporov et al. (12) and Li et al. (13) also reported Crassvirales members in the gut microbiome of rhesus monkeys, pigs, and cats.

The majority of crAss-like phages were predicted to infect bacteria in the phylum Bacteroidetes by a variety of bioinformatic methods, such as CRISPR spacer matching and co-abundance analysis (14). In addition, 17 crAss-like phages were found to link to bacteria in different genera of Bacteroidetes by the metagenomic Hi-C approach (15). To date, the isolation of crAss-like phages *in vitro* cultures has proven to be challenging (16). However, the study in the cultures of *Bacteroides intestinalis* found that crAss-like phage and bacteria could multiply in parallel due to a dynamic equilibrium between phage sensitivity and resistance through rapid phase variation of alternate capsular polysaccharides (17). In addition, a carrier state existed for crAss-like phages by delayed release or progeny from infected bacterial cells (17). These observations have implied that crAss-like phages persist in the gut in a “benign” form (8). Other studies have suggested that crAss-like phages are a part of the normal human virome. Different genera of crAss-like phages might prefer certain lifestyles and diets in humans (14). It has also been reported that crAss-like phages may be associated with a variety of diseases, such as inflammatory bowel disease (IBD) (18) and metabolic syndrome (19). However, the phylogenetic composition, genomic structure, and bacterial hosts of crAss phages in the pig gut microbiome remain largely unknown. In addition, their distribution across diverse pig populations has not yet been fully characterized.

**Figure 1:**
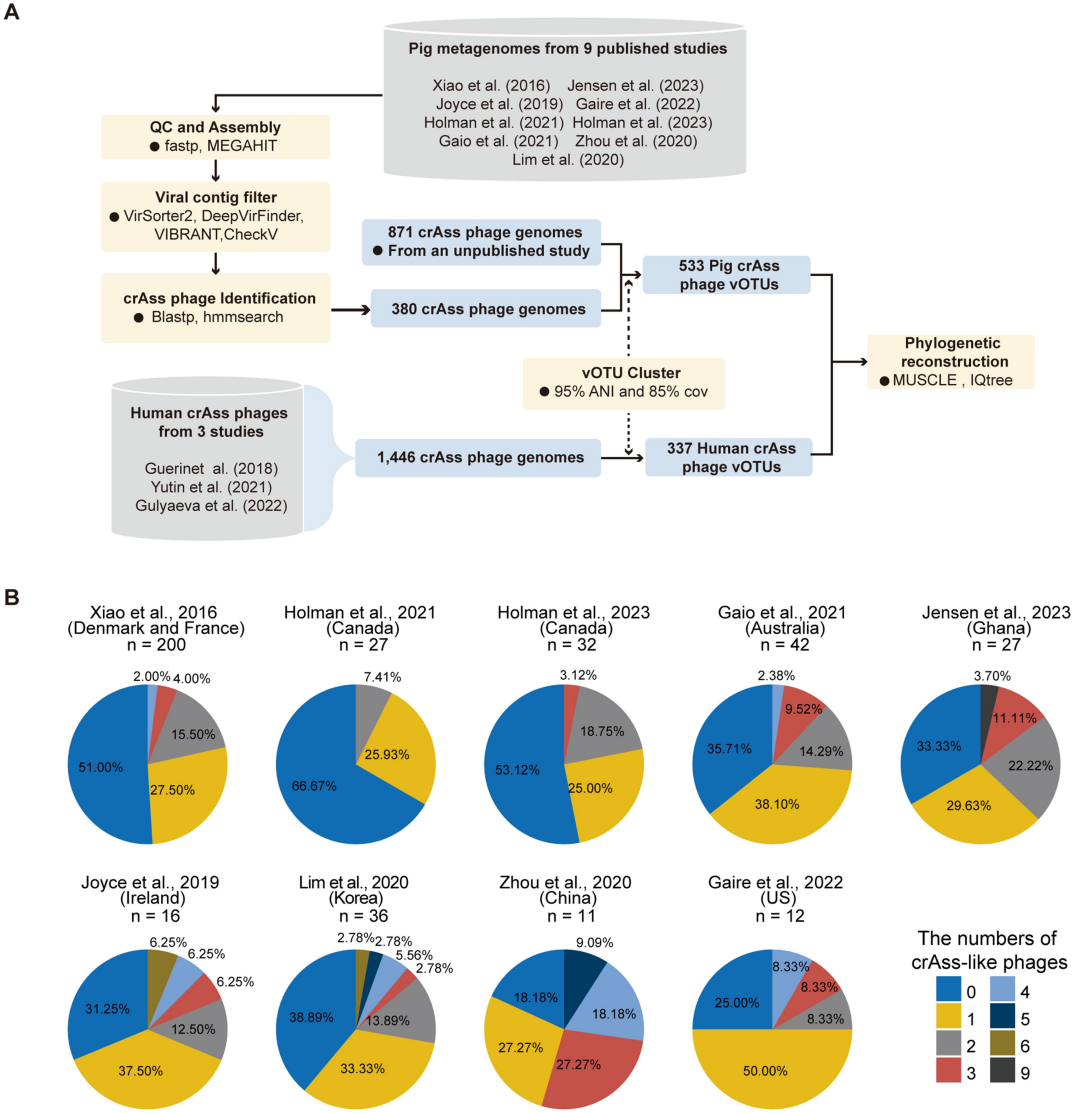
Summary of the pipeline for clustering pig crAss-like phage OTUs and the percentages of samples detected crAss-like phage genomes in each dataset. **(A)** An overview of the pipeline for clustering pig crAss-like phage OTUs. **(B)** The pie charts representing the percentage of samples in each dataset that assembled crAss-like phage genomes. The number at the top of each pie chart represents the number of samples included in each dataset.

In this study, we performed a massive study of pig gut crAss-like phages by combining (1) 380 crAss-like phages newly identified from a wide range of 403 pig metagenomes, and (2) 871 crAss-like phages previously identified in a pig gut virome dataset constructed by ourselves (Figure 1A). All these pig crAss-like phage genomes were clustered into 533 vOTUs. By constructing a phylogenetic tree based on highly conserved proteins (TerL), we found that crAss-like phages identified in pig guts were widely distributed in the clusters of Alpha, Beta, Zeta, and Delta, but were completely absent in the Gamma and Epsilon clusters. We derived and annotated 57,171 hypothetical proteins from 525 pig crAss-like phage vOTUs with taxonomic information, and found that these phages encoded numerous lysozymes and anti-CRISPR proteins. Host bacteria of crAss-like phages were also comprehensively analyzed. Based on this, we further suggested that pig crAss-like phages might interact with *Prevotella copri* affecting fat deposition in pigs. We also investigated the prevalence of pig crAss-like phages in global samples.

## 2 Materials and methods

### 2.1 The datasets of pig metagenomes and crAss phage genomes used in this study

#### 2.1.1 Pig metagenomic sequencing datasets

We collected 403 metagenomes for the identification of pig crAss-like phages from the following nine studies including PRJEB11755 (20) (*n* = 200), PRJNA526405 (21) (*n* = 42), PRJEB62878 (22) (*n* = 27), PRJEB23112 (23) (*n* = 16), PRJNA629856 (24) (*n* = 27), PRJNA857725 (25) (*n* = 32), PRJNA788462 (26) (*n* = 12), PRJEB32496 (27) (2020; *n* = 36), PRJNA647157 (28) (*n* = 11).

#### 2.1.2 Pig crAss-like phage genomes

A total of 871 pig crAss-like phage genomes were identified from the gut metagenomes of 44 pigs, including Bamaxiang pigs, Large White pigs, and wild boars from North and South of China.

#### 2.1.3 Human crAss-like phage genomes used in this study

A total of 1,446 human crAss-like phage genomes were collected from three studies and used in this study, including 249 crAss-like phage genomes identified by Guerin et al. (10), 596 crAss-like phage genomes from Yutin et al. (9) and 601 genomes from Gulyaeva et al. (18).

### 2.2 Contig assembly and identification of crAss-like phages from metagenomic sequencing data

Raw sequence reads from metagenomic sequencing data were processed to remove adaptor and low-quality sequences using fastp (v0.20.1) (29). Host contamination was further removed by mapping clean sequence reads to the pig reference genome (Sscrofa11.1) using bowtie2 (v2.4.2) (30). Clean sequence reads were assembled using MEGAHIT (v1.2.9) (31). Contigs with the length ≥ 3 kb were used for virus prediction by VirSorter2 (32), DeepVirFinder (33), and VIBRANT (34). Contigs that met one of the following criteria were retained for subsequent analysis: (1) VirSorter2 score ≥0.9; (2) DeepVirFinder score ≥ 0.9 and *p* < 0.01; (3) VirSorter2 score > 0.7, DeepVirFinder score > 0.7 and *p* < 0.05; and (4) be positive in VIBRANT prediction. Finally, we used CheckV (35) (v0.8.1) to trim bacterial contaminations in the contigs.

We used two approaches to detect crAss-like phages. First, two genetic signature proteins of p-CrAssphages (polymerase: UGP_018 and terminase: UGP_092) were queried against the protein sequences of viral genomes using blastp from the blast+ package (v2.5.0) (36). The proteins with an E-value < 1 × 10^−5^ and a query alignment length ≥ 350 bp were considered as the hits. The viruses were considered as putative crAss-like phages if its genome contained either polymerase or terminase hit and had the length greater than 70 kb. Second, identifying three conserved structural proteins (terminase large subunit, portal protein, and major capsid protein) of crAss phages in viral genomes using the hmmsearch (−E 0.001) in the HMMER package (v3.3.2) (37). We referenced the hmm profiles previously constructed by Yutin et al. (9), Since previous study has indicated the presence of alternative genetic codons in crAss phages, we also used the transeq (−clean) in EMBOSS programme (38) for translation analysis of viral sequences with six different types of open reading frames. The virus contigs were considered as candidate crAss-like phages if at least one protein sequence hit three conserved structural proteins. Finally, viral contigs identified by both methods were combined and considered as the final set of crAss-like phage genomes.

### 2.3 Clustering of crAss-like phage genomes

To construct crAss-like phage vOTUs, we combined 380 newly identified and 871 previously obtained pig crAss-like phage genomes. vOTUs were clustered at the threshold of 95% average nucleotide identity (ANI) and 85% coverage that referred to the MIUViG standard proposed by Roux et al. (39) using a custom script in the CheckV repository (35, 39). Using the same methods and thresholds, all 1,446 human crAss-like phage genomes were also clustered into vOTUs. The longest crAss-like phage genome sequence in each vOTU was chosen as the representative genomes of vOTUs.

To construct the genus-level viral clusters (VCs), we clustered 533 pig crAss-like phage vOTUs and 337 human crAss-like phage vOTUs using the vConTACT v2.0 (--rel-mode “Diamond” --db “ProkaryoticViralRefSeq201-Merged” --pcs-mode MCL - - -vcs-mode ClusterONE) based on gene sharing networks (40).

### 2.4 Phylogeny reconstruction and the assignment of genomes to crAss-like phage clusters

TerL protein sequences of 533 pig crAss-like phage vOTUs and 337 human crAss-like phage vOTUs were detected using the TerL hmm profiles provided by Yutin et al. (9). Multiple sequence alignments of TerL were performed using the MUSCLE (41) with default parameters. Phylogenetic trees were constructed using the iqtree (-B 1000) (42). The phylogenetic trees were then midpoint-rooted and visualized using the iToL (43).

Reference taxonomies of human crAss-like phage vOTUs obtained from three previous studies in humans (9, 10, 18) and expanded to pig crAss-like phage vOTUs in the phylogenetic tree. In brief, the most recent common ancestor (MRCA) of human crAss-like phage vOTUs belonging to the same taxonomy on the phylogenetic tree was determined using phangorn 2.11.1 and ape 5.6-2 in R packages. All descendants of that MRCA were assigned to the classified groups. One human crAss phage vOTU (QWCE01000387) had no reference taxonomy and did not belong to one of the six crAss phage clusters on the phylogenetic tree, so it was removed from subsequent analyses. In addition, eight pig crAss-like phage vOTUs were not annotated to any taxonomies and removed from subsequent analyses because it did not belong to the MRCA of any reference taxonomies.

### 2.5 Determination of genetic codes and tRNA scanning

The genetic codes of crAss-like phage genomes were determined using the prodigal-gv, a modified version of the Prodigal software (44). This software could automatically detect the recoding of the stop codon TAG. Detection of tRNAs in the genome was performed using the tRNA-scan-SE (2.0.12) (45) with the parameters: -G -X 35.

### 2.6 Comparison of genomic structures among different crAss-like phage clusters and functional annotation of crAss-like phage genomes

Two approaches were used to annotate the functional capacities of predicted proteins. First, protein sequences of crAss-like phage genes were aligned to the profiles of highly conserved crAss-like phage proteins constructed by Yutin et al. (9) using the hmmsearch in the HMMER package (37) with e-values ≤1e−3. And then, the unannotated proteins were further annotated using the geNomad (44) with default parameters. Proteins that could not be annotated by either method were defined as unknown proteins.

Identification of Carbohydrate-Active Enzymes (CAZymes) was performed using online dbCAN3^1^ (46). AcrPred (47) and PaCRISPR (48) were used to identify anti-CRISPR proteins (Acr) in a catalog of unknown proteins. Only those proteins identified as putative Acrs by both software were retained. Putative Acr proteins with > 200 amino acids in length were removed from further analysis because of possible false positives. The plots of genomic structure comparison were generated using ViPTree^2^ (49) with default parameters.

### 2.7 Prediction of bacterial hosts for crAss-like phages based on CRISPR spacers

Bacterial hosts of crAss-like phages were predicted by matching the bacterial CRISPR-spacer sequences to crAss-like phage genomes using blastn (−task blastn-short) (36) allowing a maximum of one mismatch across the whole spacer region sequence. CRISPR spacer sequences were predicted from two MAG datasets including the Unified Human Gastrointestinal Genome (UHGG) catalog (50) and MAGs of pig gut microbiome from our previous study using MinCED (51).

### 2.8 Determining the abundances of vOTU using metagenomic sequencing data

Clean reads of metagenomic sequencing data were mapped to the genomes of 525 pig crAss-like phage vOTUs using BWA MEM (v0.7.17-r1188) (52). The output was converted to BAM format via Samtools (v1.15.1) (53). The abundance of crAss-like phage vOTUs in a metagenomic sequencing data (one sample) was estimated by CoverM (v0.6.1)^3^ with options “contig --min-read-aligned-percent 95 --min-covered-fraction 75 --methods rpkm” and normalized the genome size and sequencing depth with RPKM (reads per kilobase per million mapped reads). Clean reads with less than 90% identity to crAss-like phage genomes were excluded from the analysis. The abundance of a vOTU in a sample was considered zero if the coverage of its genome by clean reads was less than 75% in that sample.

### 2.9 Statistical analysis

The comparisons of genome size (using complete genomes), the number of genes between marker genes (block), and the number of Acr in crAss-like phage genomes among different clusters were performed by the Wilcoxon test (pairwise comparison). The number of crAss-like phage vOTUs detected in different studies was compared by Kruskal-Wallis (multiple group comparison). Association analysis of crAss-like phage vOTUs with pig fat deposition were analyzed using the MaAsLin2 (54) in R package.

## 3 Results

### 3.1 Identification of crAss-like phages from geographically diverse pig metagenomes

To comprehensively characterize the diversity of crAss-like phages in pig guts, we collected 403 public pig metagenomes from nine studies across five continents (20–28). Using the methods described previously (see Method Details), we assembled 380 crAss-like phages from 54% of 403 tested samples (33.33–81.82% across the nine studies). The number of crAss-like phages identified in each sample ranged from one to nine (Figure 1B). To generate vOTUs of pig crAss-like phages with comprehensive representation, we also combined 871 crAss-like phages previously identified from pig gut metagenomes. These 1,251 pig crAss-like phages had an average genome size of 100.97 kb (Supplementary Table S1). At the thresholds of 85% coverage and 95% average nucleotide identity (ANI) (39), a total of 533 pig crAss-like phage vOTUs were obtained (Supplementary Table S1). More than half of these 533 vOTUs (286, 53.7%) were singleton, and the crAss-like phage vOTU containing the largest number of 51 genomes was from the metagenome of a Bamaxiang pig (BMXV030000188) (Supplementary Figure S1A; Supplementary Table S1). In order to compare human and pig derived crAss-like phages, we also collected 1,446 crAss-like phage genomes from three human studies (9, 10, 18) (Figure 1A). A total of 337 human crAss-like phage vOTUs were generated under the same threshold. The vOTU NL_crAss000703 contained the largest number of 239 crAss-like phage genomes (Supplementary Figure S1B; Supplementary Table S1). The longest available genomes of these vOTUs were selected as representative genomes and used for subsequent analyses.

All 533 pig and 337 human crAss-like phage vOTUs were combined and further clustered into 136 genus-level VCs (viral clusters) using vConTACT (v.2.0) (40). There were 31 VCs that were comprised of both human and pig crAss-like phage vOTUs, 33 VCs containing only human crAss-like phage vOTUs, and 72 VCs containing only pig crAss-like phage vOTUs (Supplementary Figure S1C; Supplementary Table S2).

### 3.2 Phylogenomics and genomic features of human and pig crAss-like phages

**Figure 2:**
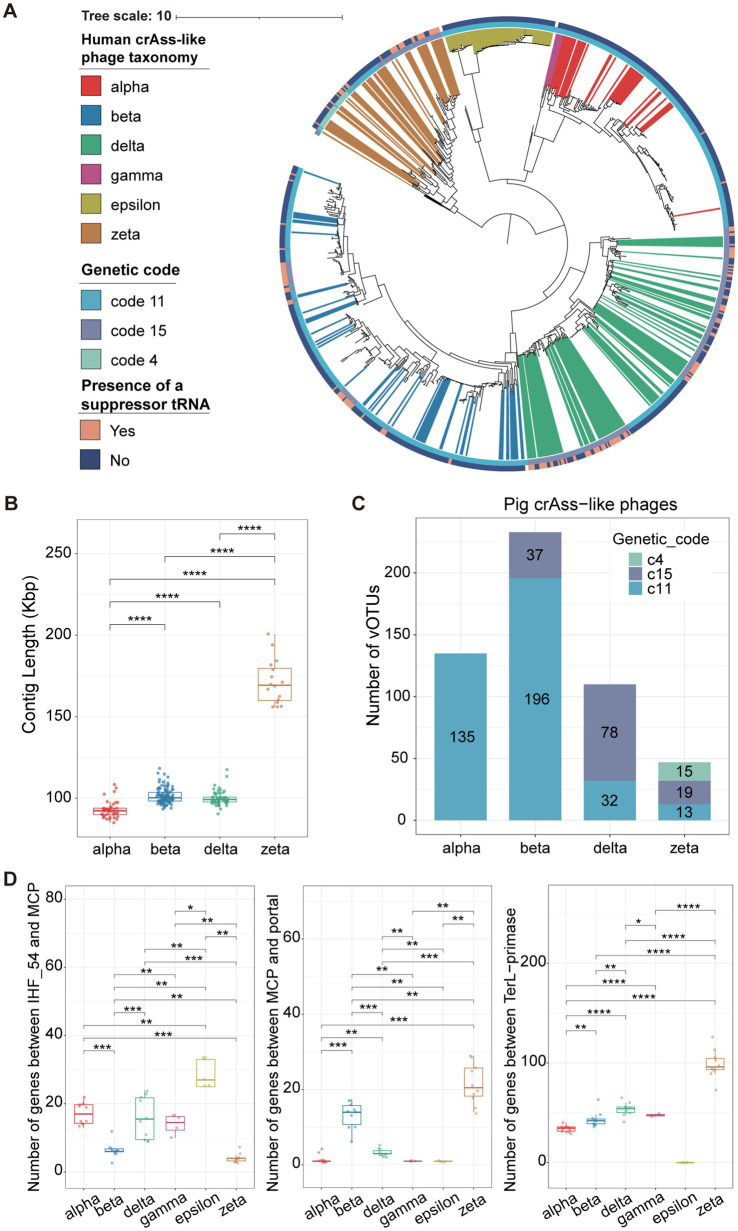
Reconstruction of the phylogenetic tree of crAss-like phage genomes and the comparison of genomic features among different crAss-like phage clusters. **(A)** A TerL-based phylogenetic tree constructed with 533 pig crAss-like phage vOTUs and 337 human crAss-like phage vOTUs. Differently colored branches represent different classifications of human crAss-like phage vOTUs at the family-level, and no colored branches represent pig crAss-like phage vOTUs. Yellow and blue colors in the outer cycle indicate the identification of suppressor tRNA or not. The different colors in the inner cycle show the utilization of alternative genetic codes. **(B)** The boxplots showing the comparison of contig lengths (genome sizes) among pig crAss-like phages from different clusters. The boxplots show medians (bold lines), the upper and lower quartiles. The comparison was performed by non-parametric Wilcoxon test. **(C)** The stacked barplots represent the number of pig crAss-like phages using different genetic codes in each crAss-like phage cluster. **(D)** The boxplots showing the comparisons of gene numbers between IHF_54 and MCP (left), between MCP and portal (middle), and between TerL and primase (right) in crAss-like phage genomes among different clusters. The number of genes between TerL and primase in the Epsilon cluster could not be determined because the primase gene was not detected in the genomes of Epsilon crAss-like phages. The boxplots show medians (bold lines), the upper and lower quartiles. The comparison was performed by non-parametric Wilcoxon test. **** *p* < 0.0001, *** *p* < 0.001, ** *p* < 0.01, * *p* < 0.05.

To comprehensively explore the evolutionary relationship between pig-derived crAss-like phages and human-derived crAss-like phages, a phylogenetic tree based on TerL (terminase large subunit) genes was constructed with 533 pig and 337 human crAss-like phage vOTUs (Figure 2A). The phylogenetic structure divided these crAss-like phage vOTUs into six distinct evolutionary branches which corresponded to alpha cluster (Intestiviridae), beta cluster (Steigviridae), gamma cluster (Crevaviridae), delta cluster (Suoliviridae), and two potential new crAss-like families (epsilon and zeta clusters) that were reported (9). Pig crAss-like phage vOTUs were widely distributed in these evolutionary branches, and 525 out of 533 pig crAss-like phage vOTUs could be classified into four crAss-like families referring to the classification of human crAss-like phage vOTUs in the same cluster using the most recent common ancestor (MRCA) approach (18). There were 135, 233, 110, and 47 pig crAss-like phage vOTUs being classified into alpha, beta, delta, and zeta crAss-like families, respectively (Supplementary Figure S2A; Supplementary Table S3). However, we did not find any pig crAss-like phage vOTUs clustered into the gamma and epsilon evolutionary branches. Just like the observation in human crAss phages (Supplementary Figure S2B), pig crAss-like phage vOTU in the zeta cluster had a significantly larger genome size (an average of 171,298 bp) (Figure 2B). Additionally, we compared the genome sizes of pig and human crAss phages within the same cluster. We found that human crAss-like phages had the larger genome size than those identified in pigs in the alpha cluster, while the reverse tendency was observed for crAss-like phages in the beta cluster (Supplementary Figure S2C).

Previous study has indicated that alternative genetic codes are widely present in human crAss-like phages (9). Using the prodigal-gv (v2.9.0), we identified a total of 149 pig crAss-like phage vOTUs using alternative genetic codes. Therein, the stop codon TAG was recoded for glutamine (genetic code 15), and the stop codon TGA was recoded for tryptophan (genetic code 4) (Figure 2A; Supplementary Table S3). Specifically, similar to the report in human crAss-like phages (9), we found no alternative genetic codes in pig crAss-like phages in the alpha cluster. Thirty-seven out of 233 pig crAss-like phage vOTUs in the beta cluster could use the genetic code 15. However, we did not find this in human crAss-like phages in the beta cluster. There were 78 pig crAss-like phage vOTUs accounting for 71% of crAss-like phage vOTUs in the delta cluster that used the genetic code 15. Furthermore, 19 and 15 out of 47 pig crAss-like phage vOTUs in the zeta cluster could use the genetic code 15 and code 4, respectively (Figure 2C; Supplementary Figure S2D). One of the common mechanisms of the stop codon reassignment is the utilization of repressor tRNAs, so we further examined the presence of repressor tRNAs in pig crAss-like phages. The results indicated that a higher percentage of pig crAss-like phages using the alternative genetic codes (26.67–94.59%) were identified with repressor tRNAs than those using the standard genetic code (0–15.38%) (Supplementary Table S3). Especially, repressive tRNAs were identified in 35 out of 37 pig crAss-like phages using the genetic code 15 in the beta cluster. Interestingly, several crAss-like phages using the standard genetic code (two, eight, and two pig crAss-like phages in the alpha, beta, and zeta cluster, respectively) also had repressive tRNAs.

To compare the differences in the genome structure of crAss-like phages between humans and pigs among six different clusters, we selected five human and five pig crAss-like phage genomes which were considered as complete genomes by CheckV (v0.8.1) from each of four clusters as representative genomes. Additionally, five human crAss-like phage genomes were selected from the gamma and epsilon clusters. We found that the positions of marker genes in the genome structures of crAss-like phages from humans and pigs in the same clusters were basically similar (Supplementary Figures S3–S7), suggesting conservation of the genome structure for crAss-like phages from humans and pigs in the same cluster. Previous studies have demonstrated that the genomes of crAss-like phages contain three large gene blocks responsible for assembly, replication, and transcription (9, 55). The gene blocks between IHF 54 and MCP, and between MCP and portal were responsible for the phage assembly, while the gene block between TerL and primase was related to replication. We compared the number of genes in each gene block among six crAss-like phage clusters. CrAss-like phages from the alpha, delta, epsilon, and gamma clusters had significantly more genes in the block between IHF54 and MCP compared to crAss-like phages from the beta and zeta clusters, but had significantly lower number of genes in the block between MCP and portal (Figure 2D). This implies differences in gene blocks encoding proteins for phage assembly among crAss-like phages from different clusters. We also found that crAss-like phages in the zeta cluster possessed significantly more genes in the block between TerL and primase (Figure 2D), which validated the previous report that indicated a complex block of replication-related genes in crAss-like phage genomes from the zeta cluster (9). The gene block for transcription did not conserve, so we did not compare its gene numbers among the six crAss-phage clusters.

### 3.3 Potential functional capacity of pig crAss-like phages

**Figure 3:**
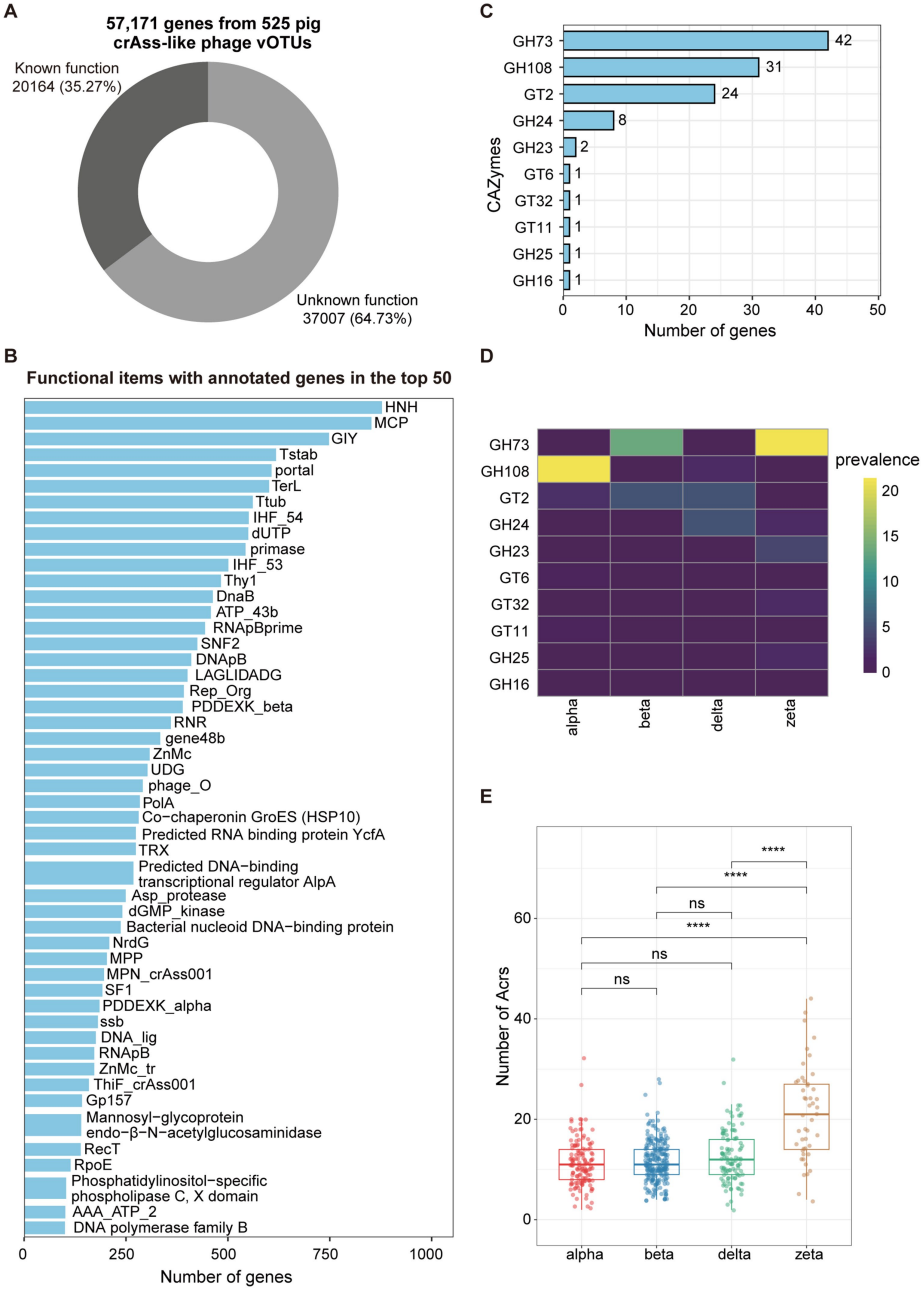
Functional capacities of pig crAss-like phages. **(A)** The pie chart representing the proportion of 57,171 genes from 525 pig crAss-like phage vOTUs that could be functionally annotated. **(B)** The barplot indicating the functional items with the number of annotated genes in the top 50. **(C)** The barplot showing CAZymes encoded by pig crAss-like phage genomes. The number of annotated genes for each item is shown on the right of bars. **(D)** Prevalence (%) of each CAZyme type detected in this study in different crAss-like phage clusters. **(E)** Comparison of the numbers of Acr detected in different clusters of pig crAss-like phages. The boxplots show medians (bold lines), the upper and lower quartiles. The comparison was performed by non-parametric Wilcoxon test. **** *p* < 0.0001, *** *p* < 0.001, ** *p* < 0.01, * *p* < 0.05.

To gain insights into the potential function capacities of pig crAss-like phages, we predicted 57,171 hypothetical proteins from 525 crAss-like phage vOTUs with family-level taxonomic information using prodigal-gv (V2.9.0). Functional annotation of hypothetical proteins was performed using the HMMsearch based on HMM profiles from Yutin et al. (9) and geNomad (44) with default parameters. Overall, 64.73% of crAss-like phage genes were not assigned to any functional items, indicating the deficiency to the understanding of functional potentials of pig crAss-like phages (Figure 3A; Supplementary Table S4). The remaining 35.27% of annotated genes mainly belonged to the functional items that conserved in crAss-like phages (9) (Figure 3B; Supplementary Table S4). For examples, HNH, one of the endonucleases that has been demonstrated to insert into some of phage genes as an intron or intronic peptide (9), contained the largest number of annotated genes. Major capsid protein (MCP), the major structural component of virus particles, had the second largest number of annotated genes (Figure 3B).

We further used dbCAN3 to predict carbohydrate-active enzymes (CAZymes) in pig crAss-like phage genomes. A total of 112 genes encoding 10 types of CAZymes were identified from 103 out of 525 crAss-like phage vOTUs, including six types of Glycoside Hydrolases (GH73, GH108, GH24, GH23, GH25, and GH16) and four types of Glycosyl Transferases (GT2, GT6, GT32, and GT11) (Figure 3C; Supplementary Table S4). GH73 contained the largest number of annotated genes (*n* = 42) and was identified in 13.7 and 21.2% of pig crAss-like phage genomes in the beta and zeta clusters, respectively. However, it was completely absent in the alpha and delta clusters (Figure 3D). GH108, which had the second-largest number of annotated genes (*n* = 31), was detected in 21.5 and 1.8% of pig crAss-like phage genomes in the alpha and delta clusters, respectively. But it was not found in the beta and zeta clusters (Figure 3D). Most strikingly, except GH16, all types of Glycoside Hydrolases encoded by crAss-like phage genes were annotated as lysozyme which can lyse bacteria by degrading peptidoglycan in the cell wall (56).

Phages have evolved a variety of anti-defense mechanisms in the “arms race” with host bacteria, such as anti-CRISPR proteins (Acr) (57). Most Acrs are short and highly variable. It was also a main characteristic that matched the large number of proteins encoded by crAss phage genes lacking annotation information. Using PaCRISPR (48) and AcrPred (47), 6,605 genes encoding Acrs were predicted from 523 pig crAss-like phage genomes, with an average of 12.6 Acrs-encoding genes per genome. CrAss-like phage genomes in the zeta cluster had a significantly higher number of Acrs than those in the other three clusters (Figure 3E; Supplementary Table S4). We speculated that this might be related to their large genome sizes and rapid sequence evolution of crAss-like phages in the zeta cluster (9).

### 3.4 Host bacteria of pig crAss-like phages and the possible effect of the interaction between crAss-like phages and bacterial species on fat deposition in pigs

**Figure 4:**
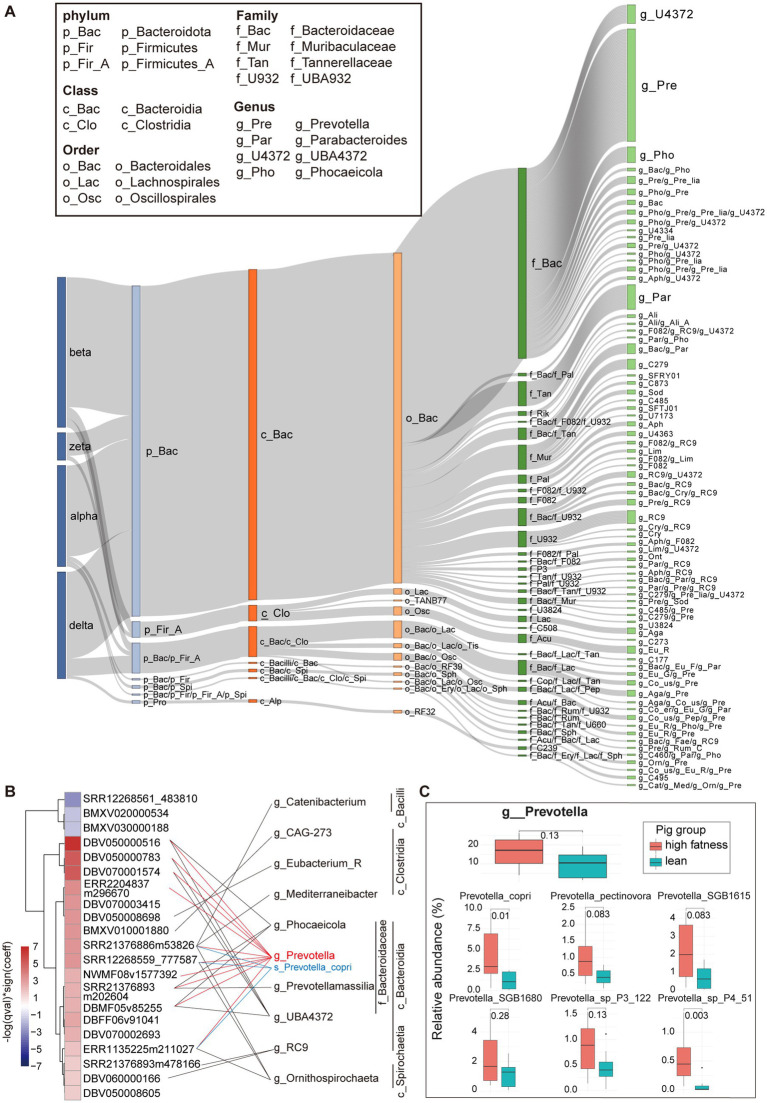
Host distribution of pig crAss-like phages and the effect of bacterial host of *Prevotella* on pig fat deposition. **(A)** The Sankey diagram representing the taxonomic distribution of bacterial hosts of pig crAss-like phage vOTUs. The colors of the rectangles represent different taxonomic levels of bacterial hosts. The length of the rectangles indicates the number of pig crAss-like phage vOTUs. The abbreviations of taxonomic names for some predominant host bacteria are shown in boxes. The abbreviations for other host bacterial taxa are only shown in Supplementary Table S5. **(B)** The heatmap (left) indicates the vOTUs associated with pig fat deposition. The fatness-associated vOTUs were identified by MaAsLin2 analysis. The purple boxes show the vOTUs negatively associated with fat deposition, and the red boxes indicate the vOUTs positively associated with high fatness. The color gradients exhibit the strength of the associations calculated by −log (qval) * sign (coeff). The bacterial hosts of vOTUs were identified through matching to CRISPR spacers. The fatness-associated vOTUs and its bacterial hosts were connected with lines. *Prevotella* and *Prevotella copri* are specially indicated with red and blue. **(C)** In comparison of the relative abundance of *Prevotella* and *Prevotella* spp. between high fatness pigs and lean pigs. The comparison was performed by non-parametric Wilcoxon test.

To explore the interactions between crAss-like phages and bacterial hosts, we extracted CRISPR spacer regions from bacterial genomes in the Unified Human Gastrointestinal Genome (UHGG) database (50) and MAGs assembled in our previous study. About 51% (*n* = 267) of 525 pig crAss-like phage vOTUs were identified bacterial hosts. Bacteroidota included the greatest number of pig crAss-like phage hosts (*n* = 229). There were 21 pig crAss-like phages whose bacterial hosts were detected in both Bacteroidota and Firmicutes A. At the genus level, *Prevotella* (*n* = 78), *Parabacteroides* (*n* = 17), and *UBA4372* (*n* = 13) contained the large numbers of bacterial hosts of pig crAss-like phages (Figure 4A; Supplementary Table S5). These phylum and genus were the predominant bacterial taxa in the pig gut microbiota (58, 59). In addition, 66.67% (*n* = 178) of pig crAss-like phage vOTUs with host information had their hosts in only one bacterial genus, whereas the other 33.33% of crAss-like phages (*n* = 89) were detected their hosts across multiple genera (≥ 2) (Figure 4A; Supplementary Table S5).

To compare the distribution of bacterial hosts between pig and human crAss-like phages, we also predicted potential bacterial hosts for human crAss-like phages. About 84.52% of 336 human crAss-like phage vOTUs (*n* = 284) were identified bacterial hosts. The taxa of bacterial hosts for human crAss-like phages were similar to that for pig crAss-like phages at all the phylum, class, order, and family levels. At the genus level, the largest number of bacterial hosts of human crAss-like phages belonged to *Bacteroides* (*n* = 59), followed by *Prevotella* (*n* = 39), and *Parabacteroides* (*n* = 23). However, as mentioned above, only 3 pig crAss-like phages were identified bacterial hosts belonging to *Bacteroides*, indicating the differentiation of bacterial hosts between pig and human crAss-like phages at the high resolution of taxonomic levels (Supplementary Figure S8A).

The interaction between phages and their bacterial hosts plays an important role in animal health or shaping animal phenotypes (60). As one of the important hosts of crAss-like phages described above, *Prevotella* has been confirmed to be associated with pig fat deposition in our previous study (61). Here we further investigated whether the interaction of crAss-like phages with *Prevotella* was associated with pig fat deposition. Sixteen fecal metagenomic sequencing data from previous study, including eight data from high fatness pigs and eight data from lean pigs (62) were used in this study. The abundances of pig crAss-like phage vOTUs were calculated by mapping clean metagenomic sequencing reads to the genome sets of pig crAss-like phage vOTUs constructed in this study. A total of 18 pig crAss-like phage vOTUs were identified to have higher abundances in high-fatness pigs, while three vOTUs showed higher abundances in lean pigs by employing the linear mixed model implemented in MaAsLin2 (Figure 4B; Supplementary Table S6). We further analyzed the host bacteria of these 21 fatness-associated crAss-like phage vOTUs and found that 14 out of the 18 vOTUs enriched in high-fatness pigs had detectable bacterial hosts. Interestingly, the host bacteria for 10 out of these 14 high fatness-associated vOTUs belonged to *Prevotella* (Figure 4B; Supplementary Table S6). Besides *Prevotella*, other bacterial genera were also identified as the host bacteria for eight high fatness-associated vOTUs. For example, five vOTUs should targeted bacteria across *Prevotella*, *Phocaeicola*, *Prevotellamassilia*, and *UBA4372* in Bacteroidaceae (Figure 4B; Supplementary Table S6). Our previous study has demonstrated that 12 *Prevotella* spp. (especially *Prevotella copri*) and three *Bacteroides* spp. were significantly associated with pig fat deposition, with higher abundances in fatness pigs (low lean meat percentage) than in lean pigs (62). *Prevotella copri* was a core bacterial species in the pig gut microbiome with high abundance. Here, *Prevotella copri* was confirmed to significantly enrich in fatness pigs (*p* = 0.01) and was identified as the bacterial hosts of three crAss-like phages which also had higher abundance in fatness pigs (Figures 4B,C). Furthermore, *Prevotella* was also enriched in high-fatness pigs, although it did not achieve a significance level (*p* = 0.13) (Figure 4C). This result was consistent with the mutually beneficial and multiply in parallel between crass-like phages and their bacterial hosts (17). This suggested that crAss-like phages in the pig gut might influence fat deposition by interactions with bacterial species in *Prevotella* and *Bacteroidaceae*. Unfortunately, we did not detect bacterial hosts for three low fatness-associated vOTUs.

### 3.5 Global distribution of pig crAss-like phages

**Figure 5:**
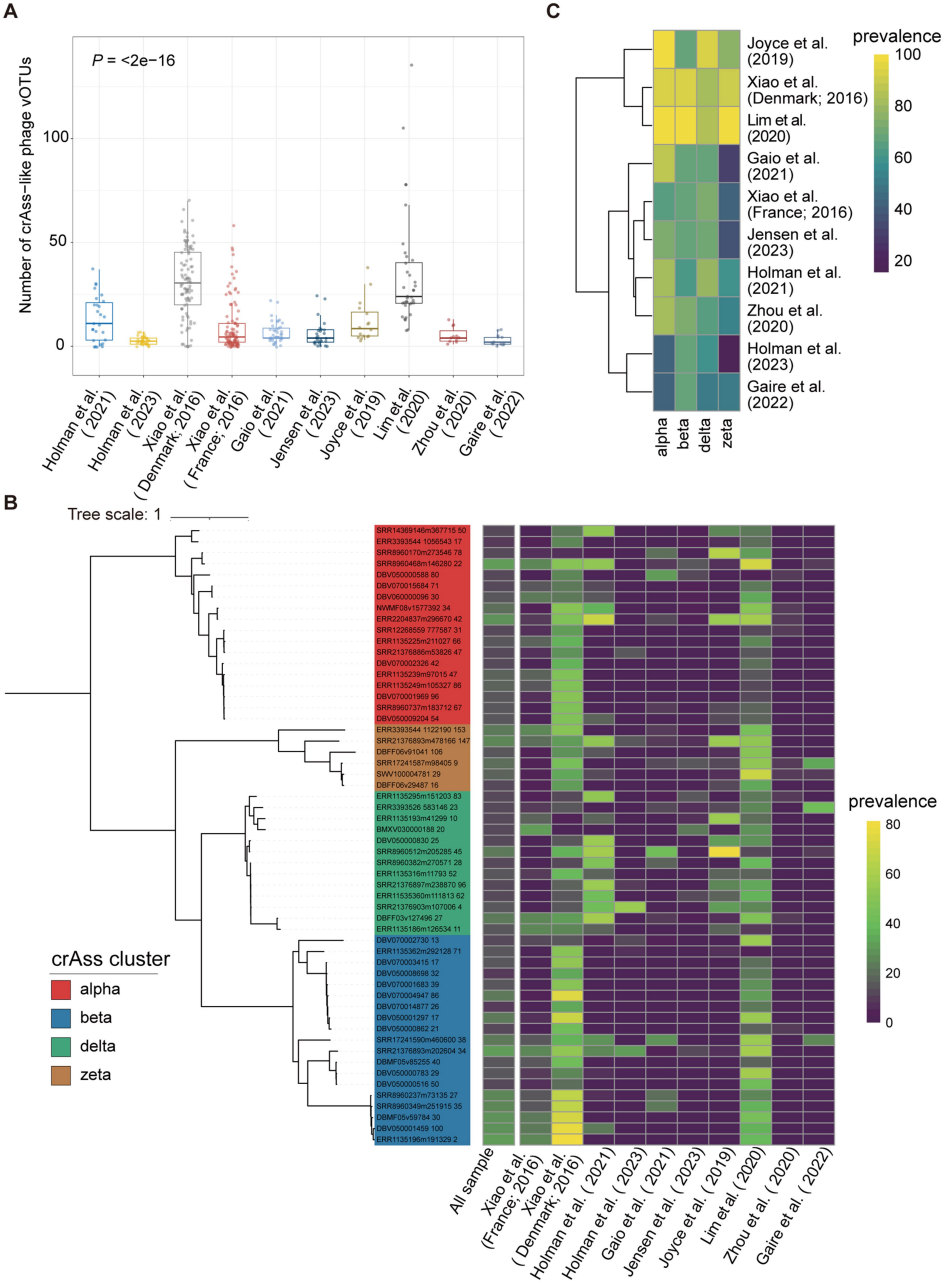
The distribution of pig crAss-like phages in geographically diverse pig populations. **(A)** The boxplots representing the number of pig crAss-like phage vOTUs identified in samples of each pig population. The boxplots show the medians (bold lines), the upper and lower quartiles. The comparison was performed by the non-parametric Kruskal test. **(B)** The TerL-based phylogenetic tree constructed with 56 pig crAss-like phage vOTUs with >10% prevalence. The heatmap on the right represents the prevalence of vOTU in all samples and in each pig population. **(C)** The heatmap representing the prevalence of four pig crAss-like phage clusters in each of 10 pig populations.

We first calculated the number of crAss-like phage vOTUs detected in each of the 403 samples from nine previous studies to assess the diversity of crAss-like phages in tested samples. A vOTU was considered to be detected in a sample if >75% of its representative genome sequence was covered by metagenomic sequencing reads from that sample. Samples from Lim et al. (27) (*n* = 36) had the largest average number of crAss-like phage vOTUs per sample detected (34.4 crAss-like phages/sample on average, ranging from 8 to 135), followed by the Xiao et al. (20) (30.9 crAss-like phages/sample). The least number of crAss-like phage vOTUs per sample was detected in Holman et al. (25) with an average of 2.8 vOTUs/sample (Figure 5A; Supplementary Table S7).

We then determined the prevalence of pig crAss-like phage vOTUs in all tested samples to identify core crAss-like phages in the pig gut. The results showed that all detected vOTUs had low prevalence. There were 56 crAss-like phage vOTUs with >10% prevalence (alpha = 18, beta = 19, delta = 13, and zeta = 6) (Figure 5B). Among them, only three crAss-like phage vOTUs showed >30% prevalence, with the highest at 30.8% (Figure 5B). The vOTU (SRR8960468m146280) with the highest prevalence belonged to alpha cluster and could target to the bacteria in *Eubacterium* and *Prevotella*. Its extensive bacterial hosts across genera likely contribute to its detection in a wide range of samples (63). However, this vOTU was not detected in samples from the studies of Holman et al. (25) and Zhou et al. (28) (Figure 5B). We further calculated the prevalence of all pig crAss-like phage vOTUs at the pig population (study) level. A vOTU was considered detected in a pig population if it was found in at least one sample of that pig population. The results showed that 119 crAss-like phage vOTUs were detected in only one pig population. Seven crAss-like phage vOTUs were detected in the largest number of eight pig populations (Supplementary Table S7).

Finally, we analyzed the distribution of four pig crAss-like phage clusters (alpha, beta, delta, and zeta) in different pig populations. A crAss-like phage cluster was considered present in a sample if any vOTUs belonging to this cluster were detected in that sample. The alpha cluster of crAss-like phages was detected in more than 80% of samples from six populations, whereas the beta, delta, and zeta clusters were identified in 80% of tested samples in only two, three, and two populations, respectively (Figure 5C). This discrepancy might be related to the fact that the alpha cluster of crAss-like phages showed the most diversity among crAss-like phages (9).

## 4 Discussion

CrAss-like phages were one of the most notable new viral members that have been widely identified in the human gut. However, limited information is available regarding their diversity, functional capacity, and genomic structure in pigs. In this study, we obtained 1,251 pig crAss-like phage genomes by assembling 380 genomes from pig metagenome data across a wide range of geographic sources and integrating 871 crAss-like phage genomes identified previously. For comparison, we also collected 1,446 human crAss phage genomes. Based on these datasets, we systematically analyzed the diversity, genomic structure, functional capacities, host bacteria, and biogeographic distribution of pig crAss-like phages. The results not only provided an important resource of crAss-like phage genomes, improved our understanding on this type of viruses, but also facilitated further studies about the interaction between crAss-like phages and bacteria, and its effect on pig health and production traits.

The phylogenetic analysis showed that, compared to human crAss-like phages, pig crAss-like phages were widely distributed in four crAss-like phage clusters (alpha, beta, zeta, and delta) reported previously, but were not found in the gamma and epsilon clusters. This might be due to the differences in the composition of gut microbiota between humans and pigs, or different living conditions for humans and pigs. It was also worth noting that this may result from the fact that our methods used for identifying crAss phages were mainly developed based on the information of known crAss phage clusters from human studies, which might be less sensitive in detecting other related clusters from pig gut microbiome. In the phylogenetic tree (Figure 2A), crAss-like phages from humans and pigs within the same cluster form a stable monophyletic group; furthermore, those within the same family exhibit a consistent gene order. This indicated that they might share a common origin or be capable of cross-species transmission between humans and pigs. Interestingly, the study by Edwards et al. (8) found that crAss-like phage strains from gorillas’ guts became closely related to humans following contact with humans.

Gene prediction using the correct genetic codes is important in the process of functional annotation, and incorrect utilization of genetic codes may lead to low predicted coding density and truncated gene products (64). Previous studies have suggested that the utilization of alternative genetic codes is a counter-defense strategy of the phages (9). Here, we found extensive utilization of alternative genetic codes in pig crAss-like phage genomes. This was consistent with the previous observations in human crAss-like phages and other types of phages (9, 65). The recoding of the stop codons could be achieved by using repressive tRNAs, which should possibly come from bacteria or point mutations in crAss phage genomes (9). We found that none of the pig and human crAss-like phages from the alpha cluster in this study used alternative genetic codes, but repressive tRNAs were identified in two pig crAss-like phage vOTU genomes. More crAss-like phage genomes from the alpha cluster should be analyzed to determine whether it was an inherent feature of the alpha crAss-like phages that they did not use alternative genetic codes.

Previous studies on crAss-like phages in humans and animals have primarily focused on conserved genes within genomes, such as key genes involved in virion assembly, replication, and transcription (9, 18). In contrast, we provide a comprehensive annotation of all functional genes in pig crAss-like phage genomes, including CAZymes and anti-CRISPR proteins. We found that except GH16, all glycoside hydrolases identified in crAss-like phage genomes were annotated as lysozyme. Previous studies have demonstrated that lysozymes encoded by phage genomes can dissolve bacteria by degrading peptidoglycan in the cell wall (56). Fujimoto et al. (66) identified an antibacterial enzyme GH25 (glycoside hydrolase family 25) LysA-like from the prophage of the highly pathogenic *Enterococcus faecalis*, which showed the potential to lyse the biofilm formed by *Enterococcus faecalis* in the gut. We also found that different types of CAZymes seem to be preferentially presented in different clusters of crAss-like phages. For example, GH73 was identified in the beta and zeta crAss-like phage genomes, but completely absent in the alpha and delta clusters. The reverse distribution pattern was observed for GH108. This implied the different lysozymes involved in the infection of crAss phages to bacteria.

The roles of crAss-like phages in health and diseases have been extensively studied. But so far, many reports have not been repeated and confirmed (8, 18). Here we found three pig crAss-like phages whose host bacteria were *Prevotella copri* which was confirmed to be causative bacterial species leading to excessive fat deposition in pigs (62, 67). Gulyaeva et al. (18) also identified *Prevotella copri* as a likely host of human delta27 crAss-like phages based on CRISPR analysis and abundance correlation. Concordance with the mutually beneficial symbiotic relationship between crAss-like phages and its bacterial hosts (17), all three crAss-like phages and *Prevotella copri* had higher abundance in high fatness pigs. Shkoporov et al. (17) reported a successful partnership between crAss001 and *Bacteroides intestinalis*, which ensured mutual persistence in complex environments. It indicated that when facing the constant selection pressures exerted by crAss-like phages, the phenotypes of *Bacteroides intestinalis* could be diversified through phase variation and point mutations. This improved the overall fitness of *Bacteroides intestinalis* and ensured its competitiveness in a constantly changing environment like the gastrointestinal tract. In addition, the persistent crAss-like phages could also potentially be involved in horizontal gene transfer that provided protection against competing bacterial strains, and gave superinfection immunity against cognate phages (17). Whether the mechanism of the interaction between crAss-like phages and *Prevotella copri* was similar to that between crAss-like phages and *Bacteroides intestinalis* or not would need to be investigated in future studies.

Finally, we found that the prevalence of crAss-like phages in pig guts was low. This suggested the heterogeneity in the composition of gut crAss-like phages. Previous study has indicated that the composition of phages in the gut was related to the composition of gut bacteria (68). We guessed that the heterogeneity in the composition of gut crAss-like phages should be caused by differential compositions of gut microbiota in different pig populations from worldwide farms.

In conclusion, our study provided 1,251 pig crAss-like phage genomes which were clustered into 533 vOTUs. This expanded the diversity of crAss-like phages. We first performed a comprehensive and systematic investigation of crAss-like phages in the pig gut, including uncovering the utilization of alternative genetic codes, annotating functional capacities of crAss-like phage genes, identifying host bacteria, and describing their distribution in broad pig populations. Although the limited sample size was used in this study, and the interactions between crAss-like phages and bacteria were inferred based on the genomic information without confirmation by experiments, this study gave deep insights into crAss-like phages in the pig gut. It also provides key resources and new knowledge for the subsequent investigation of the interactions between the virome and bacteriome in the pig gut, as well as for studies exploring how crAss-like phages affect pig health and production traits.

## Data Availability

The phage genome data have been deposited in the Zenodo repository (https://doi.org/10.5281/zenodo.13901307) without any access restrictions.

## Footnotes

^1^ https://bcb.Unl.edu/dbCAN2

^2^ https://www.genome.jp/digalign/

^3^ https://github.com/wwood/CoverM

## Supplementary material

SUPPLEMENTARY FIGURE S1 The distribution of viral contig numbers contained in each vOTU. **(A)** Viral contig numbers contained in each vOTU of pig crAss-like phages. **(B)** Viral contig numbers contained in each vOTU of human crAss-like phages. **(C)** Venn diagram indicating the numbers of viral clusters (VCs) commonly identified in both humans and pigs, and specifically identified in humans or pigs. The number of vOTUs contained in common or specific VCs are shown in parentheses.

SUPPLEMENTARY FIGURE S2 In comparison of viral contig lengths of vOTUs among six crAss-like phage clusters and between humans and pigs, and the utilization of alternative genetic codes in six clusters of human crAss-like phages. **(A)** The pie chart representing the percentage of different clades of pig crAss-like phage vOTUs. **(B)** Comparison of viral contig lengths of vOTUs among six crAss-like phage clusters in humans. **(C)** Comparison of viral contig lengths of vOTUs in six crAss-like phage clades between humans and pigs. For both **(B,C)**, the boxplots show the medians (bold lines), and the upper and lower quartiles. The comparisons were performed by the non-parametric Wilcoxon test. Since the gamma and epsilon clusters were not identified in pig crAss-like phages, these two clusters only show the length distribution of human crAss-like phages. **(D)** The stacked barplot representing the number of human crAss-like phages using alternative genetic codes in each crAss-like phage.

SUPPLEMENTARY FIGURES S3 Genomic structure plots for three gene blocks responsible for phage assembly, replication, and transcription with complete crAss-like phage genomes in the alpha cluster from pig (five) and human (five). Grey arrows represent ORFs of unknown function, light blue arrows represent ORFs of other functions, and the arrows with other colors represent marker genes as indicated in the legend at the upper of figures. The colored bands between two genomic structure plots shows the percentage of sequence identity between two genomes. The sequence identity between two genomes is also displayed in the dot plot on the left.

SUPPLEMENTARY FIGURES S4 Genomic structure plots for three gene blocks responsible for phage assembly, replication, and transcription with complete crAss-like phage genomes in the beta cluster from pig (five) and human (five).

SUPPLEMENTARY FIGURES S5 Genomic structure plots for three gene blocks responsible for phage assembly, replication, and transcription with complete crAss-like phage genomes in the delta cluster from pig (five) and human (five).

SUPPLEMENTARY FIGURES S6 Genomic structure plots for three gene blocks responsible for phage assembly, replication, and transcription with complete crAss-like phage genomes in the gramma (Figure S6A), and epsilon (Figure S6B) cluster from human.

SUPPLEMENTARY FIGURES S7 Genomic structure plots for three gene blocks responsible for phage assembly, replication, and transcription with complete crAss-like phage genomes in the zeta cluster from pig (five) and human (five).

SUPPLEMENTARY FIGURE S8 The Sankey diagram representing the taxonomic distribution of bacterial hosts of human crAss-like phage vOTUs. The colors of the rectangles represent different taxonomic levels of bacterial hosts. The length of the rectangles indicates the number of pig crAss-like phage vOTUs.
