# Supplementary figures and images for: Characterization of the diversity, genomic features, host bacteria, and distribution of crAss-like phages in the pig gut microbiome
Source: Front Vet Sci. 2025 Apr 22;12:1582122. doi: 10.3389/fvets.2025.1582122 (PMC12053484; doi:10.3389/fvets.2025.1582122)

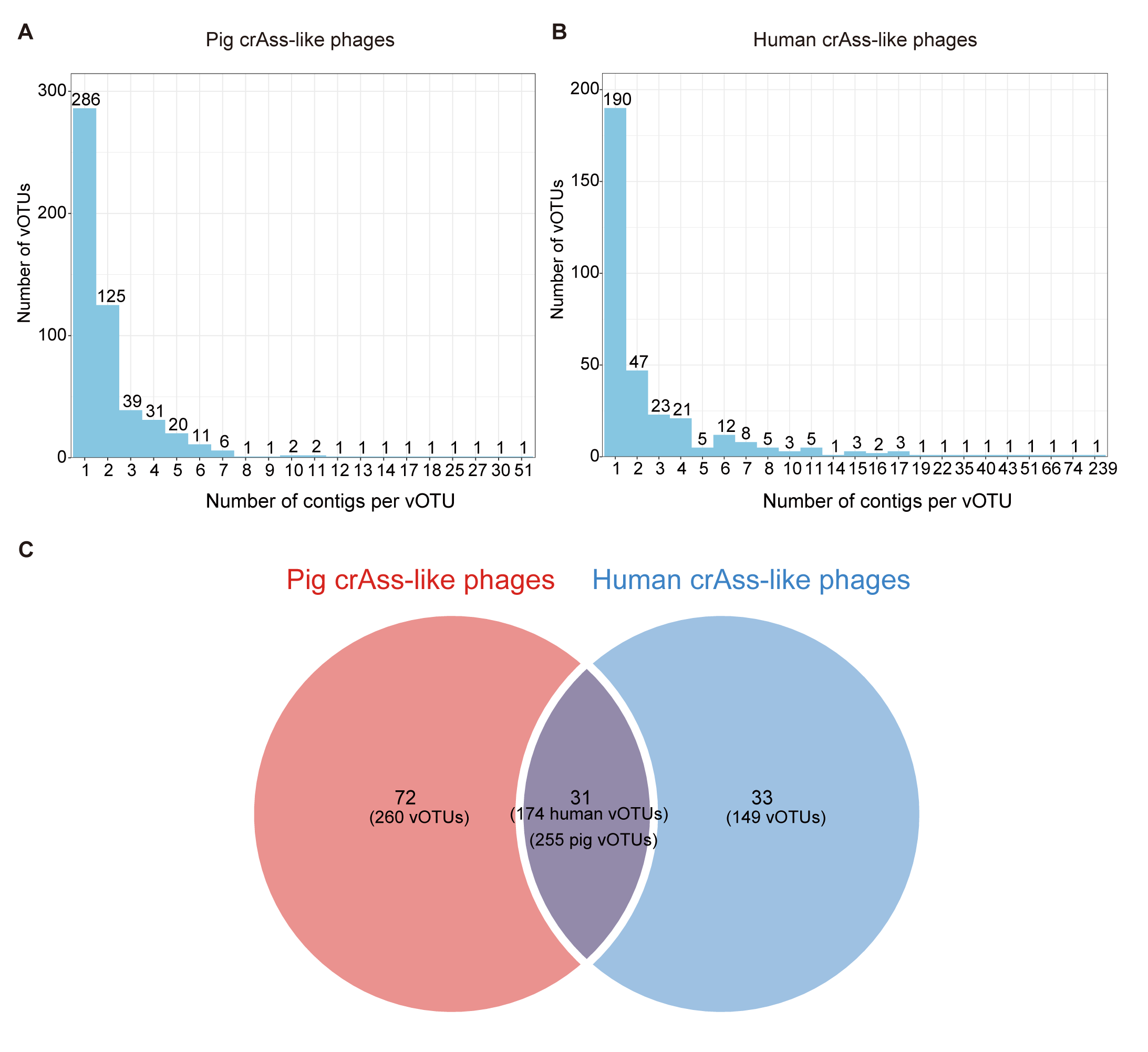

Supplement: SUPPLEMENTARY FIGURE S1 — The distribution of viral contig numbers contained in each vOTU. (A) Viral contig numbers contained in each vOTU of pig crAss-like phages. (B) Viral contig numbers contained in each vOTU of human crAss-like phages. (C) Venn diagram indicating the numbers of viral clusters (VCs) commonly identified in both humans and pigs, and specifically identified in humans or pigs. The number of vOTUs contained in common or specific VCs are shown in parentheses. [file Image_1.tif]

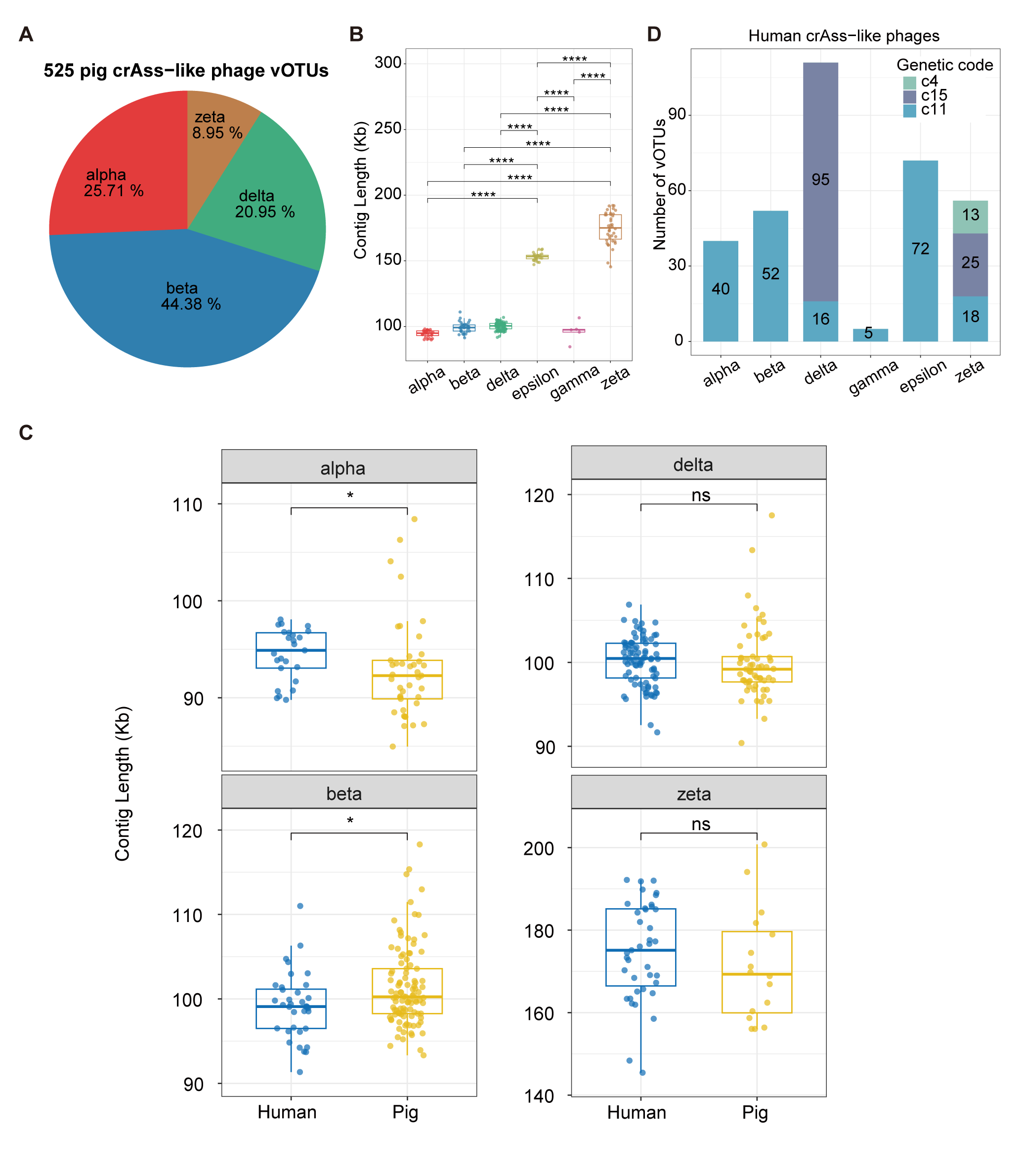

Supplement: SUPPLEMENTARY FIGURE S2 — In comparison of viral contig lengths of vOTUs among six crAss-like phage clusters and between humans and pigs, and the utilization of alternative genetic codes in six clusters of human crAss-like phages. (A) The pie chart representing the percentage of different clades of pig crAss-like phage vOTUs. (B) Comparison of viral contig lengths of vOTUs among six crAss-like phage clusters in humans. (C) Comparison of viral contig lengths of vOTUs in six crAss-like phage clades between humans and pigs. For both (B,C), the boxplots show the medians (bold lines), and the upper and lower quartiles. The comparisons were performed by the non-parametric Wilcoxon test. Since the gamma and epsilon clusters were not identified in pig crAss-like phages, these two clusters only show the length distribution of human crAss-like phages. (D) The stacked barplot representing the number of human crAss-like phages using alternative genetic codes in each crAss-like phage. [file Image_2.tif]

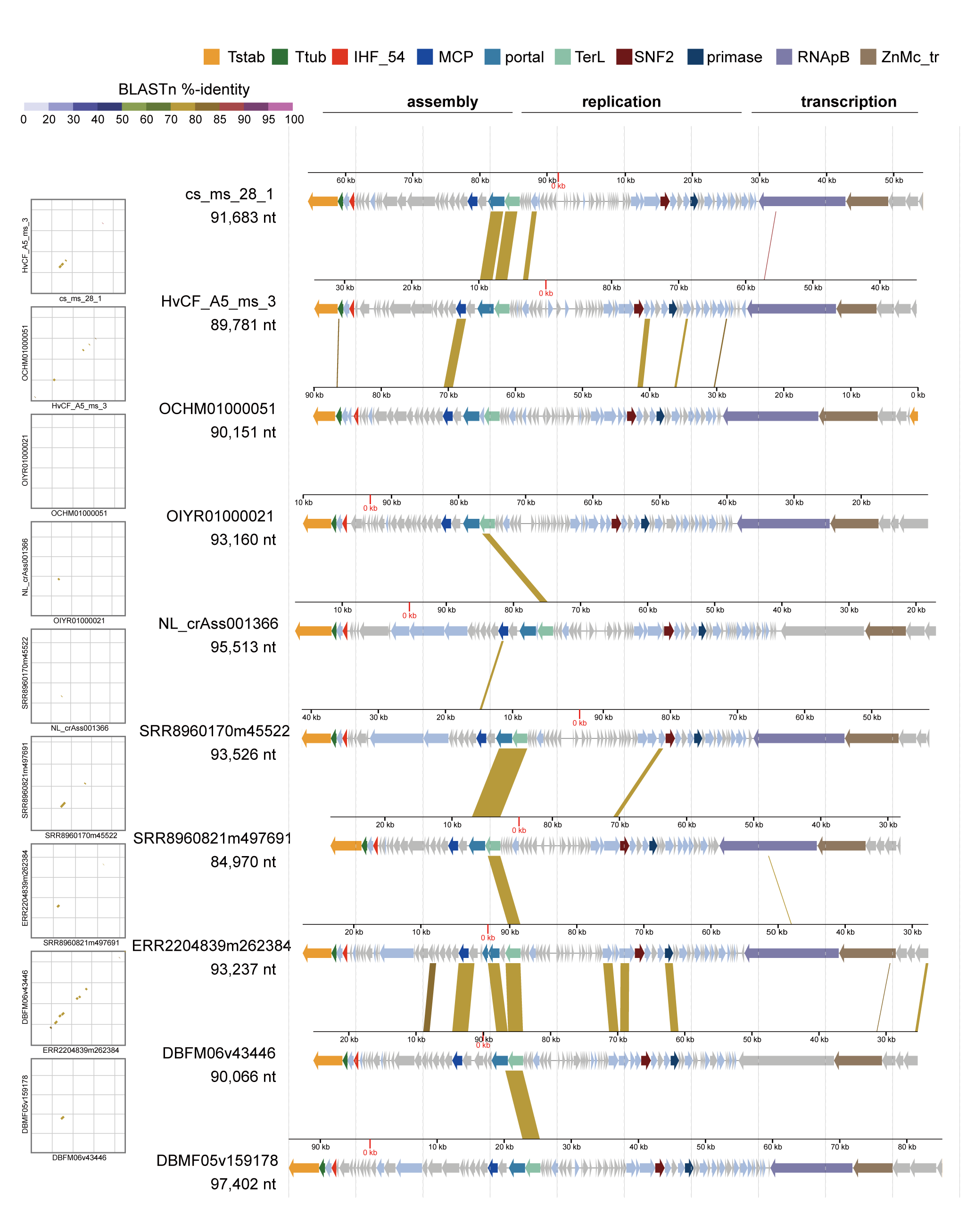

Supplement: SUPPLEMENTARY FIGURES S3 — Genomic structure plots for three gene blocks responsible for phage assembly, replication, and transcription with complete crAss-like phage genomes in the alpha cluster from pig (five) and human (five). Grey arrows represent ORFs of unknown function, light blue arrows represent ORFs of other functions, and the arrows with other colors represent marker genes as indicated in the legend at the upper of figures. The colored bands between two genomic structure plots shows the percentage of sequence identity between two genomes. The sequence identity between two genomes is also displayed in the dot plot on the left. [file Image_3.tif]

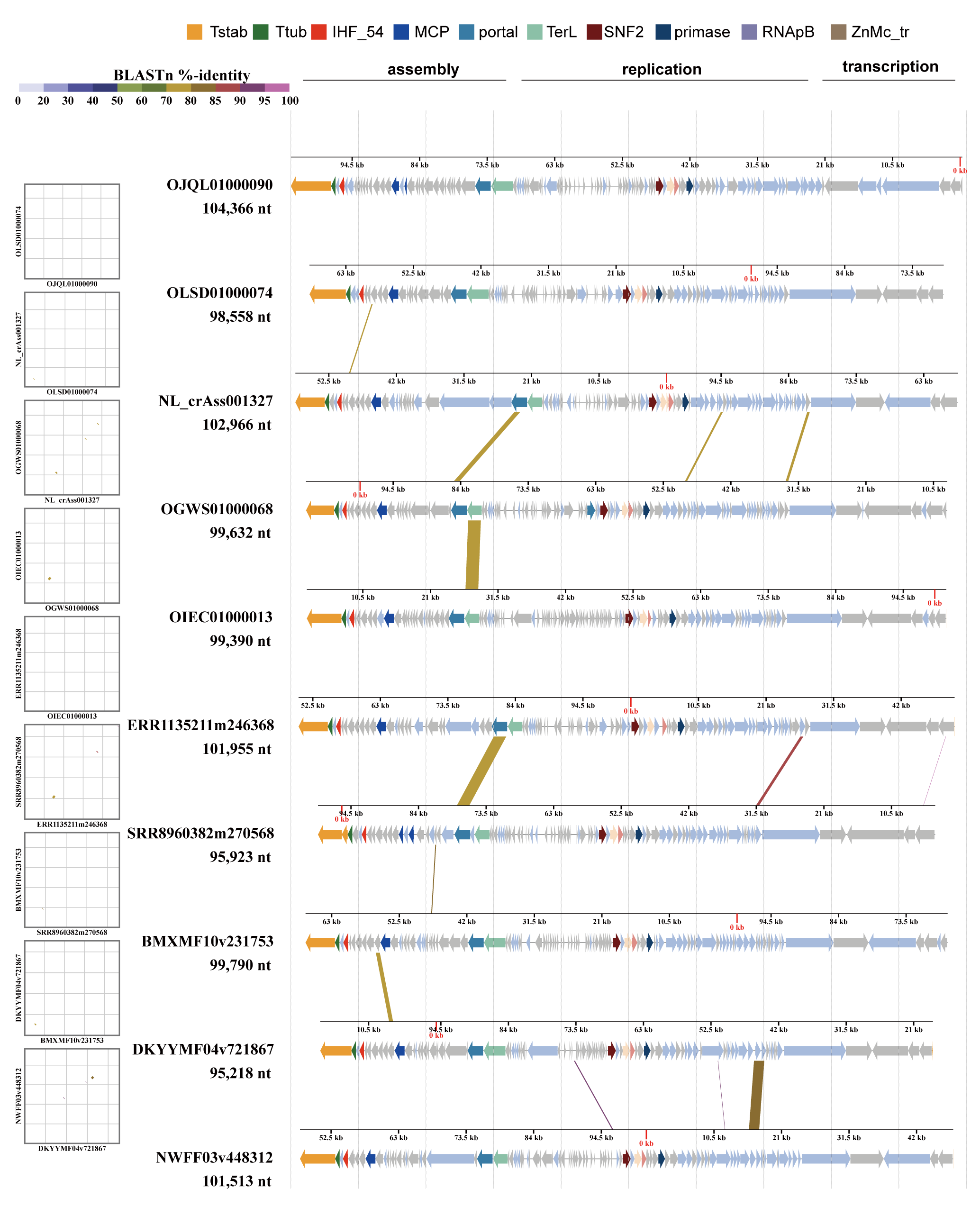

Supplement: SUPPLEMENTARY FIGURES S4 — Genomic structure plots for three gene blocks responsible for phage assembly, replication, and transcription with complete crAss-like phage genomes in the beta cluster from pig (five) and human (five). [file Image_4.tif]

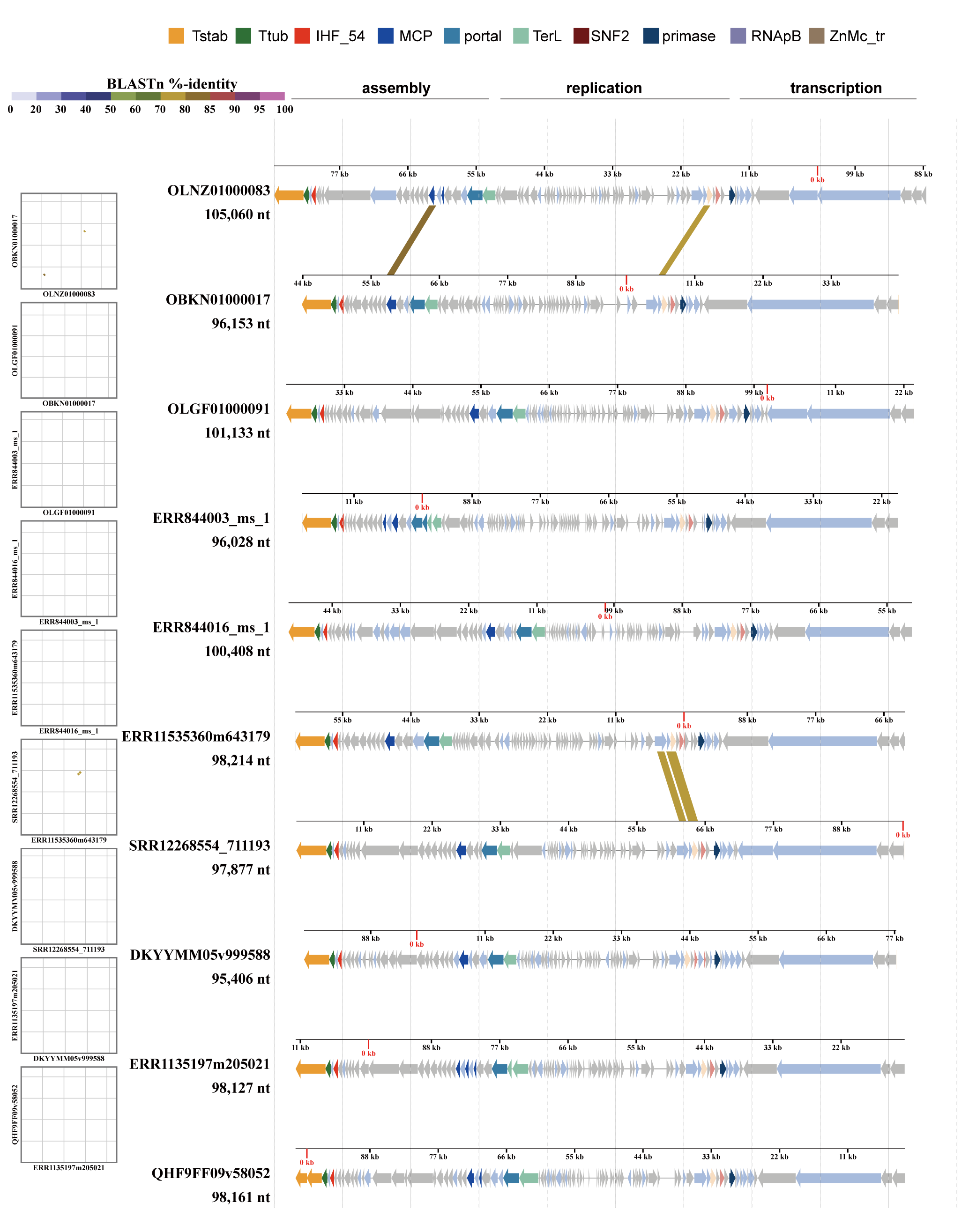

Supplement: SUPPLEMENTARY FIGURES S5 — Genomic structure plots for three gene blocks responsible for phage assembly, replication, and transcription with complete crAss-like phage genomes in the delta cluster from pig (five) and human (five). [file Image_5.tif]

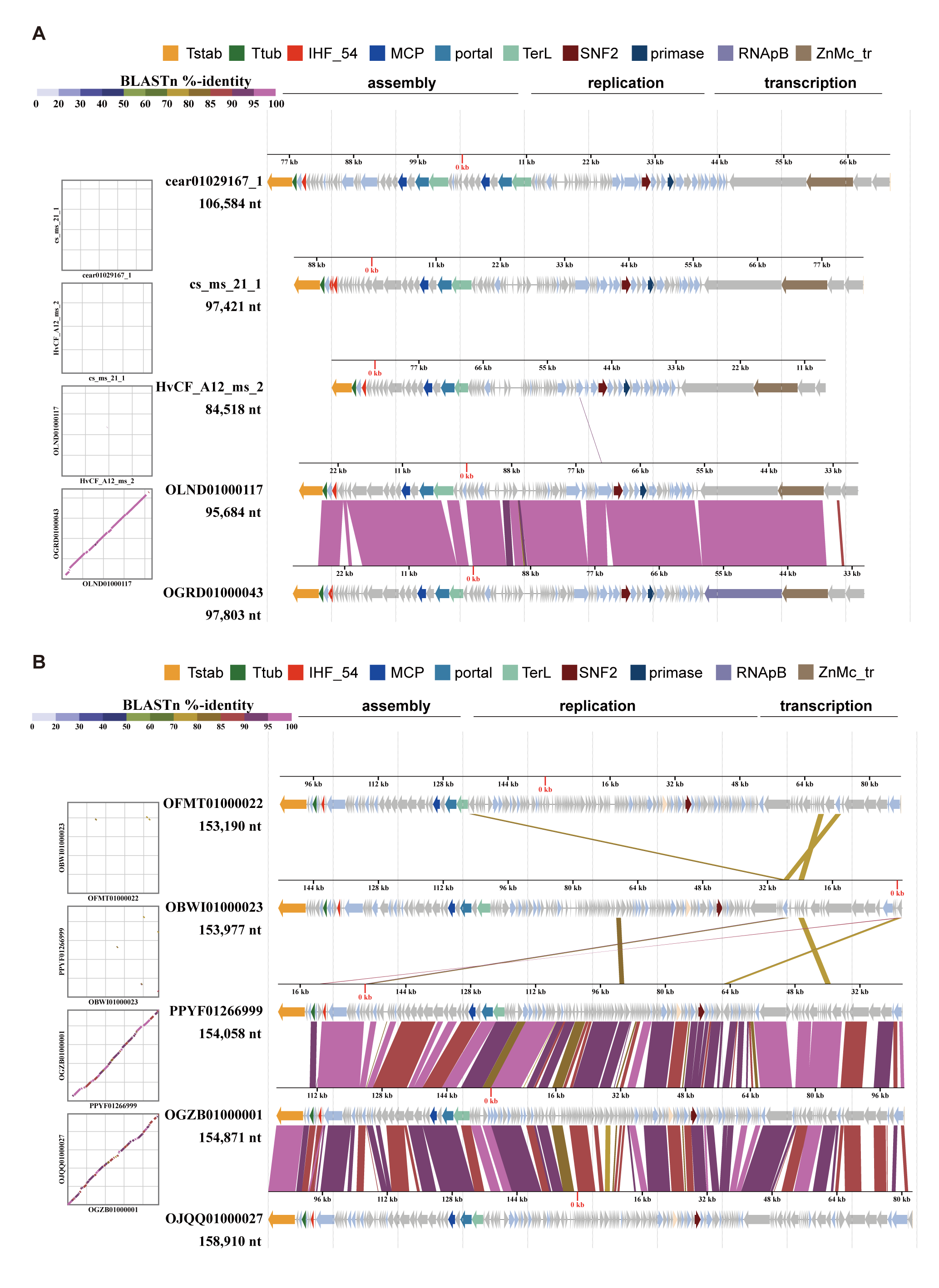

Supplement: SUPPLEMENTARY FIGURES S6 — Genomic structure plots for three gene blocks responsible for phage assembly, replication, and transcription with complete crAss-like phage genomes in the gramma (Figure S6A), and epsilon (Figure S6B) cluster from human. [file Image_6.tif]

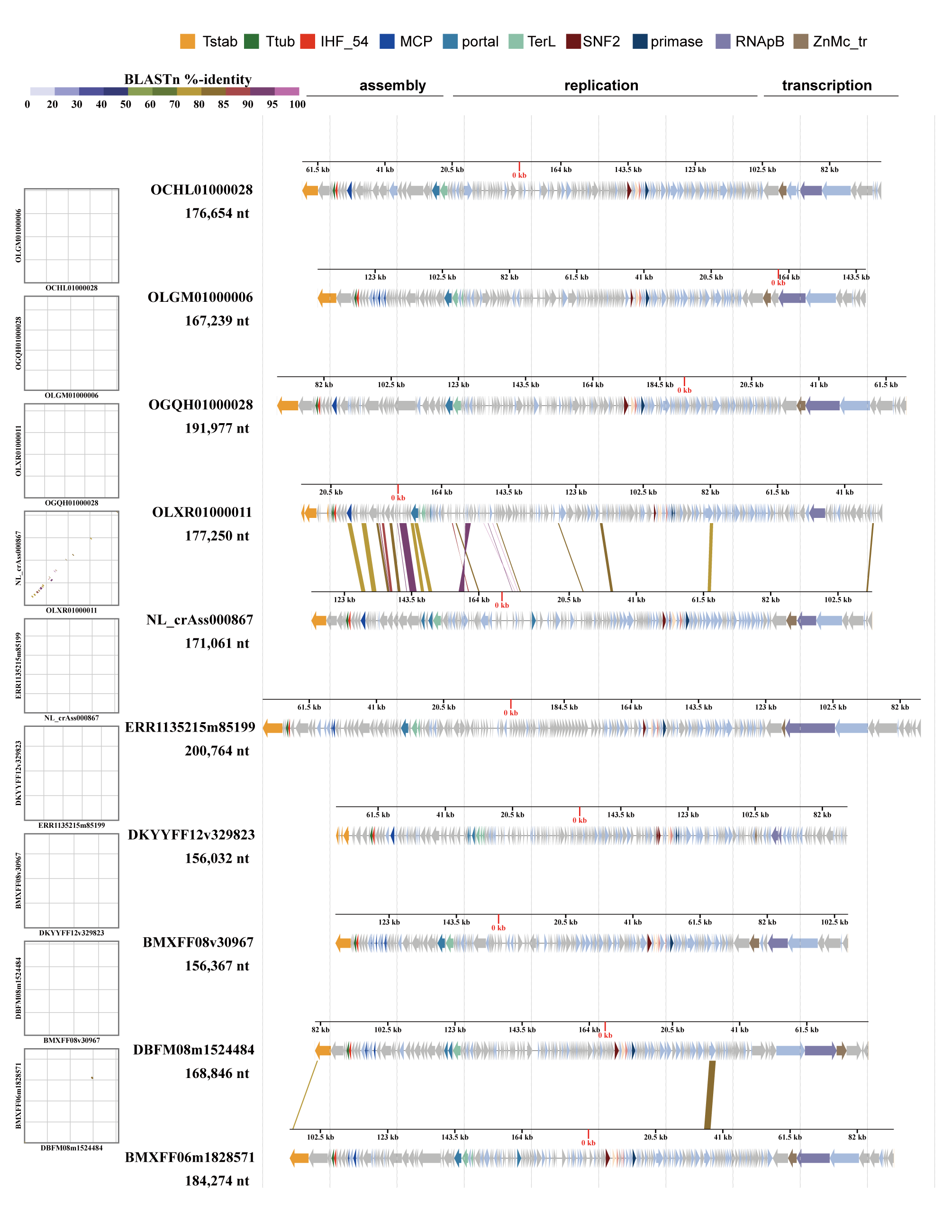

Supplement: SUPPLEMENTARY FIGURES S7 — Genomic structure plots for three gene blocks responsible for phage assembly, replication, and transcription with complete crAss-like phage genomes in the zeta cluster from pig (five) and human (five). [file Image_7.tif]

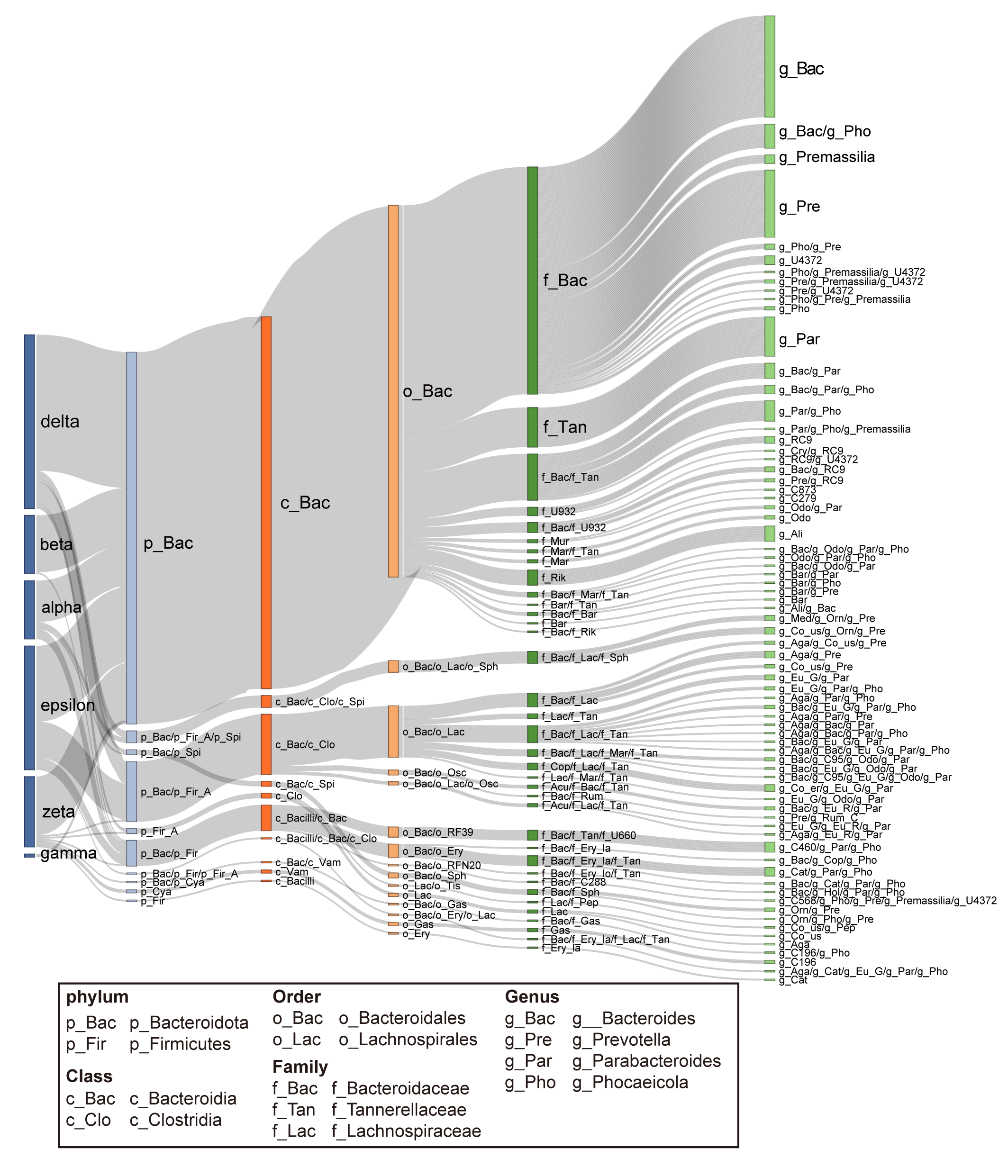

Supplement: SUPPLEMENTARY FIGURE S8 — The Sankey diagram representing the taxonomic distribution of bacterial hosts of human crAss-like phage vOTUs. The colors of the rectangles represent different taxonomic levels of bacterial hosts. The length of the rectangles indicates the number of pig crAss-like phage vOTUs. [file Image_8.tif]
